# Practitioner Adherence and Competence in MEYA, a Free Online Self-Instruction Program in Modular Psychotherapy and Counseling for Children’s Autism-Related Clinical Needs

**DOI:** 10.1007/s10803-023-06226-w

**Published:** 2024-01-26

**Authors:** Jeffrey J. Wood, Karen S. Wood, Kashia A. Rosenau, An Chuen Cho, Amanda R. Johnson, Virginia S. Muscatello, Ingrid S. Tien, Jolie Straus, Samara Wolpe, Ari Zeldin, Kristofer Kazlauskas, Bryce D. McLeod

**Affiliations:** 1 Department of Education, University of California Los Angeles, Los Angeles, USA; 2 Department of Psychiatry, University of California Los Angeles, Los Angeles, USA; 3 Department of Medicine, University of California Los Angeles, Los Angeles, USA; 4 Naval Medical Center, San Diego, USA; 5 Department of Psychology, Virginia Commonwealth University, Richmond, USA

**Keywords:** CBT, Autism, Self-instruction, Children, Youth, MEYA

## Abstract

The quality of care in public schools and other community settings for school-aged youths on the autism spectrum is variable and often not evidence-based. Training practitioners in these settings to deliver evidence-based practices (EBPs) may improve the quality of care. We developed a free internet-based training and clinical guidance system synthesizing multiple EBPs for youth on the autism spectrum addressing a range of mental health needs and autism-related behaviors, entitled *Modular EBPs for Youth on the Autism Spectrum* (MEYA; meya.ucla.edu). A multiple baseline study was conducted with seven practitioners recruited from mental health practice settings across the United States who were providing services to children on the autism spectrum (aged 6 to 17 years). Practitioners were randomly assigned to undergo baseline conditions of 2 to 8 weeks. Once online training in MEYA commenced, practitioners engaged in algorithm-guided self-instruction in EBPs for autism. Participants video-recorded sessions. Independent coders used the MEYA Fidelity Scale (MEYA-FS) to rate adherence and competence in EBPs for autism. Practitioners also completed measures pertaining to implementation outcomes and parents rated youth outcomes on personalized target behaviors. Five of seven practitioners increased their adherence to MEYA practices (i.e., MEYA-FS scores) following MEYA training. Findings for competence were similar, though somewhat less robust. Practitioners generally viewed MEYA as feasible, understandable, and acceptable. Most youth outcomes improved during MEYA. A randomized, controlled trial of MEYA would be helpful in characterizing its effectiveness for supporting practitioner EBP implementation and youth outcomes in school and community service settings.

**Supplementary Information:**

The online version contains supplementary material available at 10.1007/s10803-023-06226-w.

## Introduction

Many school-aged children on the autism spectrum receive psychotherapy or counseling in public schools and other community-based mental health settings (Stuart et al., 2017). The most common foci in psychotherapy and counseling for autistic children include disruptive and dysregulated behavior, social-communication needs, and anxiety (e.g., Brookman-Frazee et al., 2012). Unfortunately, these services are often not evidence-based (e.g., Pickard et al., 2018). One reason for this may be that few outpatient evidence-based psychotherapy or counseling interventions for children and teens on the autism spectrum have been designed for *implementation* in community settings, even though university-based efficacy studies suggest that practices like cognitive behavioral therapy are efficacious when implemented with fidelity in controlled settings (Weston et al., 2016). Methods to support practitioner implementation (i.e., user-friendly training) of evidence-based practices (EBPs) in community settings are lacking, likely contributing to the science-practice gap in psychotherapy and counseling services for autistic youth. This is unfortunate because most families do not have the financial or community resources to access private, specialty care. In contrast, public school and other community-based service settings are accessible to a diverse array of families (e.g., Martos et al., 2017; Slade, 2002). The present study entails a pilot test of a free internet-based EBP self-instruction tool (entitled *MEYA*) for practitioners working with autistic children and youth in community service settings, focusing on their implementation experiences, in an effort to assess whether larger-scale effectiveness research on MEYA may be worthwhile.

### Treatment for Autistic Youth

Autistic youth often participate in weekly outpatient interpersonal services focused on behavioral, affective, and cognitive change (e.g., psychotherapy or counseling), with the parents of nearly half of the participants in a recent services study (N = 28,009 youth on the autism spectrum) reporting at least one visit in the past year (Stuart et al., 2017). Clinical researchers have long held that intervening with both autism-related challenges and mental health needs in an integrated psychotherapy or counseling program may provide complementarity for many youths on the autism spectrum, particularly when intervention can be personalized to each child’s needs (e.g., Storch et al., 2013).

Two related efficacious evidence-based modular psychotherapy programs for autistic children have been evaluated in seven clinical trials: Schema, Emotion, and Behavior-Focused Therapy for Children (SEBASTIEN) and Behavioral Interventions for Anxiety in Children with Autism (BIACA) (e.g., Wood et al., 2020, 2021). These programs have outperformed active comparison conditions on both a priori primary outcomes (e.g., observed social engagement at school; independent evaluator-rated anxiety) as well as secondary measures (e.g., observed autism-related symptoms in the home setting; parent-reported social communication outcomes; internalizing behavior; Wood et al., 2020, 2021, 2022). Two independent replications of BIACA have been published (Storch et al., 2013, 2015).

Several features distinguish SEBASTIEN and BIACA from other evidence-based psychotherapy programs for children on the autism spectrum. These programs are modular, are tailored to the presenting needs of each child, and can address both emotion dysregulation and autism-related needs such as conversation skills, play skills, friendship development, and flexibility. Each program utilizes evidence-based behavioral practices as well as certain cognitive practices tailored to the developmental level of the client. The personalized nature of these interventions addresses the often-heterogeneous clinical needs of autistic youth. Parent and teacher consultations are incorporated for most children, and applications of practices for school and clinic-based use are provided. Further, SEBASTIEN and BIACA include design features that make the interventions accessible to autistic youth with a range of verbal communication skills. Youth motivation is emphasized using pivotal response treatment features of youth choice and shared control, embedding interests and preferred activities into sessions, rewarding attempts, and self-management (e.g., Koegel et al., 2001). In short, intervention procedures adapted for youth on the autism spectrum and delivered in a once-weekly format are efficacious in controlled research. They have the potential to contribute to effective practice in public schools and other community treatment settings. In this study, building from the multifaceted EBPs comprising the SEBASTIEN intervention, we developed an interactive internet-based training and clinical guidance application for practitioners entitled *Modular EBPs for Youth on the Autism Spectrum* (*MEYA*; Wood & Wood, 2017).

### Barriers and Solutions to the Implementation of EBPs in Community Service Settings

Research suggests that complex EBPs require reliable training and quality control procedures for successful implementation (Dolcini et al., 2021). However, if costly training and supervision procedures are needed to implement such interventions, this could limit efforts to improve the quality of care for autistic youth in many community settings (Brookman-Frazee et al., 2012; Wood et al., 2015). Thus, a critical question is how to achieve successful training outcomes through cost-effective, scalable means.

An emerging consensus in the field of implementation science is that the most economical and scalable approach for training practitioners entails remote instruction facilitated by accessible technology (i.e., internet/video) (Fairburn & Patel, 2014; Kazdin & Blasé, 2011). Internet-based EBP training platforms have the potential to be flexible and may rely primarily on algorithm-driven self-instruction or may be supplemented with limited additional remote training/supervision procedures (e.g., brief consultation calls) depending on cost–benefit analyses and available resources. An internet-based training and clinical guidance platform focused on EBPs for children on the autism spectrum could provide community-based practitioners with the means to develop expertise with relevant EBPs in a scalable and cost-effective manner.

Remote training approaches have been rated favorably by some participants (e.g., Worrall & Fruzzetti, 2009) and have been found to be a cost-effective method to increase knowledge (Heck et al., 2015; Sholomskas et al., 2005). However, when rigorous assessment methods are used, some internet-based training programs are only slightly more effective at improving practitioner knowledge than reading written materials not delivered online (Miller et al., 2004; Sholomskas et al., 2005). Studies have illustrated the need to consider alternatives to multiple-hour videotaped versions of dyadic training within internet-based training programs (often 6 to 12 h of the treatment developer speaking about the clinical intervention). Comparatively, Dimeff and colleagues (2009) used sophisticated online training procedures adapted for delivery via internet. Findings illustrated an advantage for online training, with the highest effect sizes in knowledge, competence, and integrity at post and 90-day follow-up in comparison to written materials or workshop training.

A potential implication from the research on online practitioner training suggests the adaptation of training methods aligned to the context, with shorter windows of time to devote to training in comparison with in-person training programs. Accordingly, the self-guided, internet-based training and clinical guidance platform for community-based practitioners designed in this study, MEYA, used brief training segments accessed throughout an intervention with a client, rather than a long upfront “workshop” style training before intervention utilization. This *just-in-time* training feature was one of several principles comprising our practitioner training model for MEYA (see Online Resource 1). The present study utilized a multiple baseline design with masked observational ratings of practitioners’ adherence and competence to preliminarily evaluate practitioner uptake and experience in using MEYA.

## Methods

### Participants

Participants included seven practitioners conducting psychotherapy or counseling with a child or teen on the autism spectrum. All participants—practitioners and children—lived in the United States. Practitioner eligibility was determined through an initial screening phone call in which study information was given and eligibility criteria were assessed. Initially, 11 practitioners indicated an interest in the study, and 10 consented to participate. However, three practitioners discontinued the study due to their inability to find eligible children to include, leaving seven practitioners who completed measures and participated in the primary study procedures. Table 1 and Online Resource 1 Table 1 present descriptive information for participating practitioners and children.

**Table 1:** Practitioners’ demographic characteristics and training/licensure status

Variable	*n* (%)
Gender (female)	6/7 (85.7%)
Ethnicity	
Latino/a	1/7 (14.3%)
Caucasian	6/7 (85.7%)
Degree/discipline	
Psy.D	1/7 (14.3%)
Ph.D	1/7 (14.3%)
MFT	2/7 (28.6%)
MSW	1/7 (14.3%)
MS	2/7 (28.6%)
Licensed^a^	5/7 (71.4%)
Years as a Therapist	*M* = 8.14, *SD* = 6.23 (range: 3–20)

^a^Two graduate students studying clinical psychology were included in this study

Practitioners were recruited through the Autism Speaks Autism Treatment Network, medical centers, regional centers, parent support groups, and schools; study flyers were used in recruitment. A university-based institutional review board approved the study. Contact was initiated by practitioners to the study coordinator, who conducted an initial phone screening. Practitioners who qualified and gave written consent then provided study information to families referred to them. Practitioners and parents gave written informed consent, and children assented to participate after receiving a complete description. Practitioners received gift cards worth $250 for completing study measures and interviews. Parents and children received gift cards worth $100 (split equally) for participating in assessments. The study was administered remotely from the university with which the first author is affiliated.

Eligibility criteria for practitioners included working within a recognized field of practice (e.g., clinical psychology, counseling, social work) and providing services to some youth on the autism spectrum. Eligibility criteria for youth participants included being between 6 and 17 years of age with a clinical diagnosis of autism and being enrolled in outpatient intervention with a participating practitioner. To maximize external validity, no other restrictions were imposed, although the characteristics of the child participants were assessed (see Online Resource 1 Table 1 and 2). The principal investigator reviewed the eligibility criteria for each child participant to determine eligibility status. Participants were notified of their eligibility status by the study coordinator. A detailed description of the practitioners’ background is provided below; participants have been given code numbers for this article to protect their identity.

### Practitioner Background

Practitioners provided intervention for autistic children and youth in one or more of the following settings: schools, community mental health agencies, university-based specialty clinics, private practice, and training clinics for graduate students. Participants were queried about their licensure, training and practice background, and experience working with autistic children in open-ended interviews. Not all participants provided complete information with regard to these background characteristics; all available information is summarized forthwith (also see Table 1).

Participant 1 has an M.A. in Marriage and Family Therapy (MFT; licensed and certified in a state in the western US), is a Board-Certified Behavior Analyst (BCBA), and is a Registered Play Therapist (RPTS). Participant 1 defined her theoretical orientation as primarily behavioral, with a secondary orientation in attachment theory. This participant reports working with between two to three hundred autistic children, including delivering classroom and school consultations.

Participant 2 has a Ph.D. in Psychology and has extensive experience conducting CBT, including for individuals on the autism spectrum. This participant identifies her theoretical framework as CBT, including exposure and response prevention (ERP) and mindfulness-based cognitive therapy. She is licensed in psychology and board certified in CBT.

Participant 3 is licensed as a pediatric psychologist and has a doctoral degree (Psy.D.) in School Psychology and two master’s degrees, one in Clinical Counseling and the other in Educational Psychology. He is also a licensed, doctoral-level board-certified behavior analyst. The participant describes his theoretical orientation as behavioral, utilizing many applied behavior analysis (ABA) strategies. He has practiced for 13 years.

Participant 4 is a Licensed Clinical Social Worker (LCSW) who has six years of experience working with children on the autism spectrum. She identifies her theoretical framework as CBT and Socio-Dramatic, Affective Relational Interventions (SDARI).

Participant 5 has a background in ABA and a master’s degree in Clinical Psychology, with an emphasis in Marriage and Family Therapy. Participant 5 describes her theoretical orientation as behavioral and CBT. She is a Licensed Marriage and Family Therapist and has experience providing psychotherapy to children of all ages for the past five years.

Participant 6 is a graduate student in Clinical Psychology, has a master’s degree in Psychology, and is in her third year of doctoral training. This participant has a background in CBT and parent management training.

Participant 7 is also a graduate student. She has a master’s degree in Psychology along with some experience with ABA in an in-home setting with autistic children. This participant describes her theoretical framework as CBT and dialectical behavior therapy (DBT).

### Procedures and Randomization

Once a family consented for the child to participate in the study along with the participating practitioner and completed the initial assessment (see below), the practitioner was randomized to one of four non-concurrent baselines: 2, 5, 6, or 8 sessions in length. Randomization was conducted by the last author using a computerized randomization table. This investigator had no contact with participants. The study coordinator was informed of the baseline length assignment for each participant by the last author and subsequently notified participating practitioners about their baseline assignment.

#### Baseline Phase

At the beginning of baseline, psychotherapy/counseling commences as per the practitioner’s typical intervention approach. Treatment-as-usual continues for all designated sessions of the practitioner’s baseline phase. All sessions are videotaped via smartphone, tablet, or webcam and live-streamed to a secure server for coding by the research team (see below).

#### MEYA Phase

At the end of the baseline period, each practitioner is given a login/password combination to the MEYA website (meya.ucla.edu; Wood & Wood, 2017). At that time, practitioners are asked to view a 2-h initial training video on the MEYA website and to begin using MEYA to guide module selection and content for their ongoing sessions. MEYA is described below. Practitioners are asked to complete and record at least eight sessions with the child participant during the MEYA phase, unless psychotherapy/counseling is completed earlier. During the MEYA phase, practitioners are also offered two brief consultation phone calls (10 to 20 min each) with the second author, a clinical expert in EBPs for autistic children and youth. In these consultations, feedback regarding interventions and use of the website are addressed at the practitioner’s discretion. Practitioners are also asked to continue to video-record each session during the MEYA phase. While practitioners are implicitly encouraged to use the MEYA training platform, given the study’s focus, they have autonomy to decide whether to use it or not; there are no reminders or prompts.

MEYA assumes a standard once-weekly outpatient psychotherapy or counseling format. An important feature of MEYA from the practitioners’ standpoint is that most of the training in specific clinical interventions is delivered in a just-in-time format so that practitioners learn or refresh their knowledge of the relevant EBP immediately (e.g., hours or days) before the session in which the skill is to be implemented, consistent with the training model outlined in the introduction of this paper. In the typical utilization of MEYA for session preparation, the practitioner logs into the MEYA website to select the intervention module for the upcoming session and receives brief video-vignette based training in the specific EBP. Then they carry out the session when they meet with the child and repeat the process for the next session.

### Intervention: MEYA Features and Implementation

Because this is the first article published on MEYA, its rationale and functionality are described in detail. The self-instructional features of MEYA were designed based on principles of adult learning and user-centered design (UCD) (e.g., Lyon & Koerner, 2016); see Online Resource 1 for a discussion of these principles. MEYA modules are comprised of multiple EBPs found to be probably efficacious for autistic children and youth, integrated into standard 50-min sessions (see Online Resource 1 Table 3 for a list of specific EBPs utilized in MEYA). Reviews of EBPs for children on the autism spectrum (e.g., Odom et al., 2010) and evidence-based psychotherapy programs for autism (e.g., Danial & Wood, 2013) were primary sources of EBPs considered for SEBASTIEN (Wood et al., 2021), and, thus, MEYA. Ultimately, EBPs that were complementary, that were relatively simple to learn, and that addressed multiple sources of resilience (e.g., child’s skills and attitudes; caregivers’ warmth and behavior support skills; the school and community context) were selected and composited into deliverable sessions. MEYA modules address six broad clinical foci: disruptive and dysregulated behavior, negative affect (anxiety and depression), rigid and repetitive behavior, peer engagement in school and the community, conversation and friendship, and self-care skills. All modules include sessions specifically for in-school usage (e.g., teacher consultation) and most sessions vary examples between school-based and clinic-based implementation of the session content. Adaptations for children with minimal expressive language and different levels of cognitive development are offered in most sessions in MEYA. MEYA was developed iteratively with ongoing feedback from practitioners (see Online Resource 1).

MEYA aims to help practitioners to (a) implement the target EBPs with fidelity and (b) select among the modules for specific youth. Two features of MEYA help achieve these objectives: (a) self-instructional components, and (b) an algorithm used to select modules.

#### Self-Instructional Training Design Features in MEYA

First, 16 brief (2–5 min) videos of EBPs were developed by reviewing session recordings from the SEBASTIEN clinical trial (Wood et al., 2021) and writing scripts based on the conversational flow, topics, and some paraphrased quotes from these sessions. A professional video production company, PluckStudio, LLC, created video enactments of the MEYA EBPs based on these scripts in collaboration with the first and second authors. These videos are embedded in the MEYA website in each module (e.g., demonstrating the implementation of graded exposure in the anxiety/depression module) as a supplement to the text, providing access to modeling of skills and multi-modal training materials.

Second, consistent with our aim of giving practitioners a small number of distinct choices within the intervention structure to enhance motivation and engagement, most modules have several *session types*. For example, the *Anxiety and Depression* module has four session types: exposure therapy, reframing, behavioral activation, and school consultation. Each MEYA module gives practitioners general guidance on how many sessions (in ranges) of a given session type would typically be needed to achieve clinical benefit or to determine that the session type was not a good fit for a child or youth at present. However, practitioners use their clinical judgment about the specifics of these decisions. The emphasis of this design feature is to provide session content alternatives that practitioners can select based on their clinical style, case formulation, and the child’s response to the varying EBPs.

Third, consistent with the emphasis on *just-in-time* learning to support practitioners’ recall and fluency, almost all training content in MEYA is packaged in brief self-instructional lessons aimed at supporting the practitioner’s preparation for a single upcoming psychotherapy or counseling session: in addition to the videos mentioned above, there are step-by-step instructions for the use of relevant EBPs for each type of session (e.g., an exposure therapy session within the anxiety and depression module); a practitioner “cheat sheet” for each session type; sample cartoons to use in working with the child, and parent and youth handouts for each session type. An auto-scoring self-quiz and a mental rehearsal prompt are provided for each MEYA session to encourage practitioners to engage with the material sufficiently before each upcoming session. It was assumed that preparation for each session using these materials would take 20 to 60 min for practitioners who had not reviewed them before. The only preparation suggested before beginning this session-to-session training format entails watching a 2-h initial training video, which provides practitioners new to MEYA with an overview of EBPs for school-aged children on the autism spectrum and guidance on use of the website.

Fourth, our model was influenced by the user-centered design (UCD) (e.g., Lyon & Koerner, 2016) emphasis on lowering the cognitive load and simplifying complex training materials (e.g., from the original intervention manual). Some of the simplifications of SEBASTIEN content included shortening practitioners’ step-by-step instructions and rewriting them for clarity and simplicity, omitting some practices that were partially redundant with other practices, and scaffolding critical but complex EBPs with technological enhancements, thereby helping practitioners use the techniques properly. For example, one key enhancement in MEYA is an app (the *MEYA Chart*) that allows practitioners and families to select relevant weekly goals for an incentive program for the child, which can be printed or emailed as a pre-formatted chart. Relatedly, a repository of sample goals is provided for each module to assist practitioners and families in selecting appropriate weekly goals and using positive, child-friendly wording, which can auto-populate into the MEYA Chart when selected. Lastly, a critical method for simplifying intervention planning in the self-instructional context is the Session Selector/Planner, which assists practitioners in identifying well-suited EBPs to learn and use with a particular child to a specific target of intervention; this is described next.

#### Repeated Assessments of Goal Attainment/Problem Reduction

A major feature of MEYA is its Session Selector/Planner, which is on the practitioner’s homepage and provides module recommendations for each session for each child based on an underlying algorithm (explained below). This feature is driven by an initial personalized assessment of the child/youth’s top goals or problems across the six clinical areas, which is conducted in the first session. Because children on the autism spectrum can have difficulty with self-awareness regarding their emotions and how others perceive their behaviors, this assessment was conducted with caregivers as a semi-structured interview derived from the Youth Top Problems measure (YTP; Weisz et al., 2011). This interview (Wood et al., 2017) elicits up to two high-priority goals or problems for each of the six clinical areas addressed in MEYA from the caregivers’ perspective (in their own words, e.g., “hits other children while waiting at the bus stop”). During the interview, caregivers rate each problem in terms of its severity on a 0–10 Likert-type scale, with a 10 being a *very, very big problem*. This assessment generates up to 12 youth top problems (YTPs), representing goals or challenges to address in the intervention. Caregivers are then asked to rate these weekly during the intervention, serving as weekly input data for the Session Selector/Planner algorithm.

The Session Selector/Planner recommends a module before each intervention session for each child based on an algorithm using the most recent caregiver ratings on their child’s set of YTPs. The algorithm works as follows:

1. It first recommends at least one session of two “core” modules: (1) Introduction to self-management and perspective-taking for children; and (2) Introduction to goal setting with incentives for parents.
2. Then it recommends the module for the first clinical area with a current YTP score of at least 5 out of 10 in the following order of priority: (1) disruptive and dysregulated behavior, (2) anxiety and depression, (3) rigid and repetitive behavior, (4) peer engagement in school and the community, (5) conversation and friendship, and (6) self-care skills.
3. The module for the next highest-priority clinical area with a score of at least 5 is recommended to the practitioner if: (a) the YTPs for the first recommended clinical area are rated 4 or lower, (b) there is at least a 50% reduction on the YTP scores for the first clinical area compared to baseline, or (c) the maximum number of sessions in the first recommended clinical module has been reached (this varies according to module).
4. The system continues to recommend modules accordingly until no YTPs have a score above 4 or the maximum number of sessions across all relevant modules has been reached.

The algorithm’s output is a tag on the practitioner’s homepage next to a specific module indicating that it is the *Recommended Module* for the upcoming session. In keeping with the emphasis on choice, practitioners are free to select any module they wish to use for any session, regardless of which module is recommended.

### Measures

#### Child and Youth Characteristics

Several measures were administered at baseline to assess children’s clinical and developmental characteristics. The Social Responsiveness Scale 2 (SRS-2; Constantino, 2012) is a 65-item parent-rated scale that was administered to assess the extent of the child’s autism-related challenges (e.g., social awareness, preoccupations) (Constantino, 2012). The SRS-2 has high internal consistency and accurately distinguishes children with and without autism. The WISC-V is a general ability test for youth 6 to 16 years old with established reliability and validity (Wechsler, 2014). In this study, the *Vocabulary* subscale was administered to children over the phone to help characterize their oral language skills (all children were native English speakers).

#### Youth Clinical Needs

The Youth Top Problems scale (YTP) is a valid and reliable personalized symptom assessment method that is sensitive to treatment response in youth (Weisz et al., 2011), including those on the autism spectrum (Wood et al., 2022). The YTP semi-structured interview developed for MEYA (Wood et al., 2017, described above), was administered at baseline; parents were asked to state in their own words what problems were the most concerning to them for each of the six clinical areas. Interviewers then obtained severity ratings for each problem on a scale ranging from 0 (*not at all*) to 10 (*very, very much*). A caregiver rated the two highest-rated problems or goals for each clinical area before each session (each child could therefore have up to 12 problems or goals rated for their weekly personalized assessment).

#### Practitioner Adherence and Competence in MEYA

The Modular EBPs for Youth on the Autism Spectrum Fidelity Scale (MEYA-FS; see McLeod et al., 2022) was designed to assess adherence and competence for practices found in EBPs for autistic youth. Parallel items for adherence and competence are used to assess universal elements common to many EBPs (e.g., assigning out-of-session tasks) and several core practices articulated in programs like SEBASTIEN (e.g., perspective-taking training, exposure). Coders watch entire sessions and rate each item on adherence and competence. For adherence, coders rate items on a 7-point extensiveness scale (Hogue et al., 1996) with the following anchors: 1 = *not at all*, 3 = *somewhat*, 5 = *considerably*, 7 = *extensively*. For competence items, coders rate items on a 7-point scale with the following anchors: 1 = *very poor*; 3 = *acceptable*; 5 = *good*; 7 = *excellent*. Competence ratings were only made when the practice was delivered in a session.

The present study uses the *Change in Adherence* and *Change in Competence* subscales from the MEYA-FS, which were developed to document practitioner change in the use of and expertise in EBP packages for autistic youth over the course of clinical utilization. It can be challenging to estimate treatment fidelity in modular programs because practitioners can deliver some, or all, of the modules based on the client’s presenting problem. As such, traditional scoring approaches that rely on a simple average of all items would underestimate adherence and competence, since some modules may not be delivered. The *Change* subscale scores are designed to address this problem by providing estimates of adherence and competence to the MEYA program regardless of the modules that the practitioner delivers over the course of a treatment episode. In the measure development study (McLeod et al., 2022), the *Change* subscales exhibited good interrater reliability and moderate session-to-session and inter-practitioner variability and were statistically significant predictors of treatment outcome in SEBASTIEN, in the expected direction. The *Change* subscales comprise the mean of four clusters of items, each represented by the maximum score on the 1 to 7 scales noted above from a pool of specific items. The first cluster comprises common practices used in EBP packages for children and youth on the autism spectrum like SEBASTIEN (i.e., Modeling, Rehearsal, Cognitive, In-Session Reinforcement). The second cluster includes two items relevant to the social-communication component of autism (i.e., Perspective Taking, Peer Skills). The third cluster comprises two items representing in-session behavioral procedures (i.e., Self-Management, Exposure). The fourth cluster consists of three items representing methods for assigning intervention tasks outside of the session to promote generalization (i.e., Goal Chart, Home Based Rewards, Homework). In summary, high scores on the *Change* subscales represent fidelity to relatively universal components of EBPs used in MEYA (Wood et al., 2020, 2021). For this sample, the average inter-rater reliability, ICC (2,2), was 0.79 and 0.70 for the *Change in Adherence* subscale and the *Change in Competence* subscale, respectively.

#### Implementation Outcomes

Two measures were used to assess implementation outcomes, the Usage Rating Profile-Intervention (URP-I; Briesch et al., 2013), and a quiz for practitioners (MEYA Content Quiz) developed for this study. Three URP-I subscales were used: Acceptability (12 items; i.e., satisfaction with various elements of MEYA), Understanding (8 items; i.e., mastery of MEYA concepts), and Feasibility (8 items; i.e., suitability for use; practicability). The URP-I was administered post-training and for this sample, alphas were 0.95, 0.70, and 0.67 for the Acceptability, Understanding, and Feasibility subscales, respectively. The MEYA Content Quiz comprises 20 items assessing practitioners’ knowledge of MEYA concepts and has a multiple-choice format. It has a score range of 0 to 20. It was administered at baseline, after the completion of the first three sessions, and again at the end of the training period.

### Coding and Session Sampling Procedures

Three doctoral students in clinical psychology comprised the MEYA-FS coding team (100.0% female; *M*_age_ = 25.34 years, *SD* = 0.58; 100.0% White). During coder training, a combination of didactic instruction, review of the scoring manual, review of sessions with the trainer, and coding exercises were used to promote understanding of each item. Then, coders engaged in independent coding and discussed the results in weekly meetings. Finally, each coder independently coded 30 sessions, and reliability was assessed. To be certified for independent coding, each coder had to demonstrate “good” reliability on each item (ICC > 0.59; Cicchetti, 1994). The coding order was determined by random assignment. Each session was double-coded. Coders were naïve to study hypotheses.

### Data Analysis

Visual analyses and phase-related nonoverlapping frequency tabulations of the multiple baseline practitioner adherence and competence data were conducted, followed by linear mixed modeling (LMM) of the same data using SPSS software (Version 28) (Ferron et al., 2009). LMM is a full-information analytic procedure that provides relatively accurate interval estimates of the treatment effect in multiple baseline studies using the Satterthwaite method coupled with an autoregressive level 1 error structure (Ferron et al., 2009). LMM models were fit for the MEYA-FS scales, with fixed effects for study phase [i.e., baseline vs. MEYA (coded 1 and 0, respectively)] and random effects for intercept and treatment effect. Due to the emphasis on within-practitioner change in this study, individual item scores were group-mean centered around their baseline mean for each practitioner before being aggregated into the *Change in Adherence* and *Change in Competence* subscales.

## Results

All children in the sample had an SRS-2 total T-score of 68 or greater (*M* = 80.7; see Online Resource 1 Table 1), reflecting significant autism-related challenges (Constantino, 2012). Practitioners successfully recorded most sessions, although occasionally failed to do so or experienced technical difficulties preventing recording, leading to a small number of gaps in the data [6 of 76 (7.9%) baseline or MEYA sessions were not recorded].

### Adherence

**Fig. 1:**
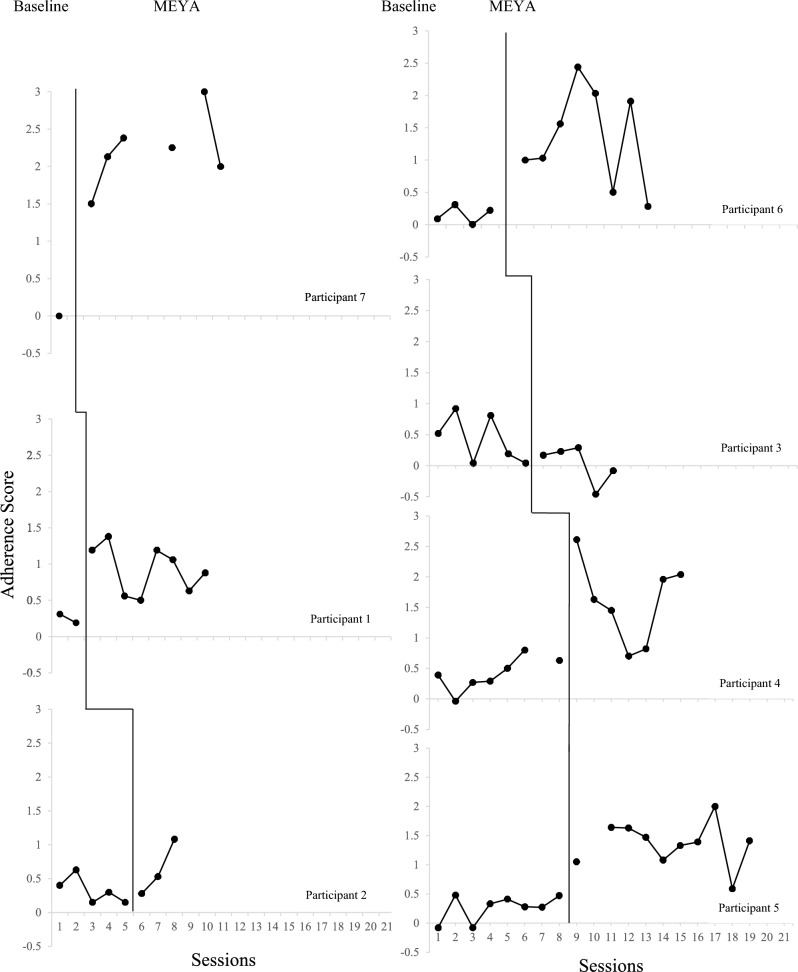
Individual participant MEYA-FS change in adherence scores for the baseline phase and the MEYA phase

Visual analysis suggests that five of the seven participants increased their adherence to MEYA clinical techniques, with nonoverlapping data in all (Participants 1, 5, and 7) or all but one (participants 4 and 6; 85.7% and 87.5%, respectively) sessions following the onset of their access to the MEYA website (see Fig. 1), in comparison to their highest *MEYA-FS Change in Adherence* score during the baseline period (see Online Resource 1, Table 4, for the session-by-session adherence data for each participant). Conversely, one of the seven participants (Participant 3) exhibited a notable reduction in their *MEYA-FS Change in Adherence* scores after the baseline period (no nonoverlapping data). Based on this pattern of scores, it is possible that Participant 3, who had been practicing ABA and related behavioral techniques for years, was already using many relevant EBPs during baseline, and yet struggled to recalibrate the delivery and competence of variations of these practices in MEYA that differed from his typical practice. One other participant (Participant 2) showed minimal change in their *MEYA-FS Change in Adherence* scores following baseline [1 of 3 MEYA sessions (33.3%) were coded as higher in adherence than their highest baseline *MEYA-FS Change in Adherence* score]. There was a trend of a positive upward trajectory for Participant 2 just as the MEYA sessions were terminated.

This pattern was reflected in the LMM for *MEYA-FS Change in Adherence* scores. The initial model, as described in the Data Analysis section above, yielded a final Hessian matrix that was not positive definite (i.e., there was a failure of model convergence). However, there was a statistically significant treatment effect in this model. The random effect of treatment was removed, and the simplified model converged without error. In Table 2, coefficients for this model are summarized, showing that there was a statistically significant positive effect for phase (i.e., the onset of MEYA training following the baseline period) in which participants’ *MEYA-FS Change in Adherence* scores increased once the use of the MEYA website began. This statistical test supports the interpretation of the graphical data discussed above. To further probe this model, exploratory LMMs were used to test for intervention effects on individual *MEYA-FS Change in Adherence* items. Overall, participants’ scores on 7 of 10 items exhibited statistically significant improvement (*p*s < 0.05) from baseline to the start of MEYA training: participants’ scores increased on the Modeling, Rehearsal, In-Session Reinforcement, Perspective Taking, Peer Skills, Goal Chart, and Home-Based Rewards *MEYA-FS Change in Adherence* items once MEYA training began. Participants’ scores did not increase significantly on the Cognitive, Self-Management, and Exposure items. The Homework item had insufficient variance for an LMM to converge.

**Table 2:** LMM models for effect of MEYA versus baseline on practitioner adherence and competence

Fixed effect	Coefficient	Standard error	*t*-ratio	*p-*value
Intercept, *γ*_*00*_	0.36	0.17	2.14	0.064
MEYA,* γ*_*10*_	0.86	0.13	6.86	< 0.001
Random effect	Variance component	Standard error		
Residual, *e*	0.28	0.05		
Intercept, *r*_*0*_	0.14	0.10		
Fixed Effect	Coefficient	Standard error	*t*-ratio	*p-*value
Intercept, *γ*_*00*_	0.03	0.16	0.17	0.871
MEYA,* γ*_*10*_	0.39	0.16	2.52	0.014
Random effect	Variance Component	Standard error		
Residual, *e*	0.37	0.07		
Intercept, *r*_*0*_	0.08	0.07		

N = 7. The fixed effect of MEYA represents the increase in adherence or competence once access to MEYA training was provided to a participant following their baseline phase

### Competence

**Fig. 2:**
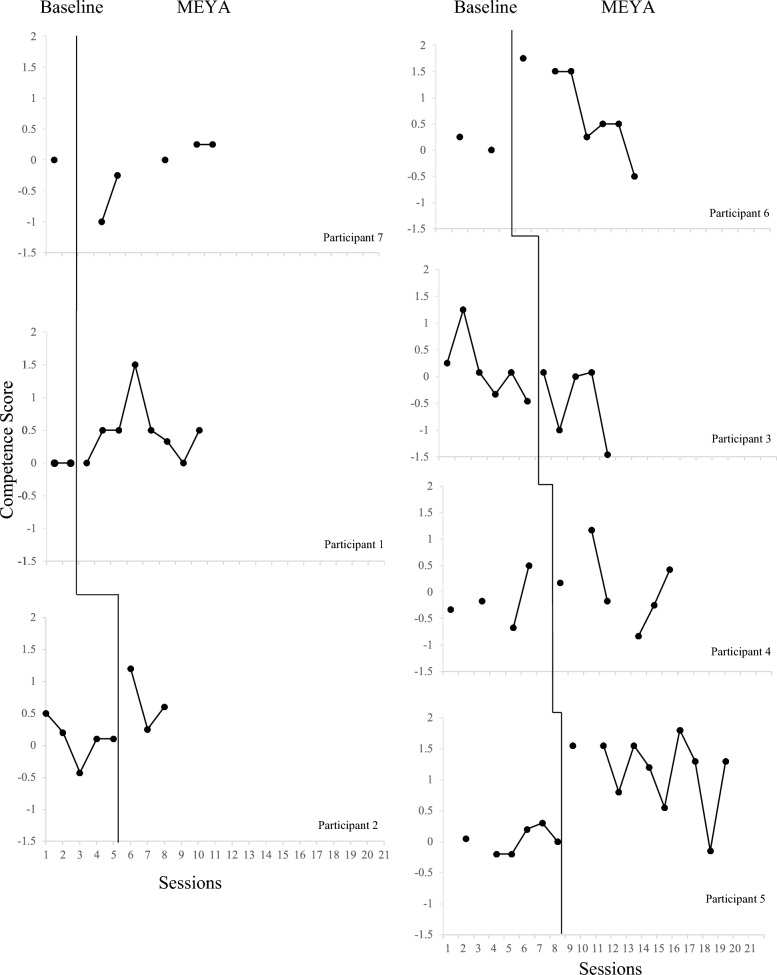
Individual participant MEYA-FS change in competence scores for the baseline phase and the MEYA phase

Plotted data for *MEYA-FS Change in Competence* scores are presented in Fig. 2. Also see Online Resource 1, Table 5, for the session-by-session competence data for each participant, as the visualization of data in Fig. 2 is small and can make it difficult to determine whether certain data points were nonoverlapping or not. There were numerous missing competence scores (compared to the more complete data for adherence as depicted in Fig. 1) because competence is only coded when a particular MEYA practice is utilized. Hence, several baselines were sparse, and score patterns were somewhat less distinct than with the more complete adherence data. Nevertheless, visual analysis suggests a positive effect of the MEYA phase on *MEYA-FS Change in Competence* scores for at least four of the seven participants: 1, 2, 5, and 6. These participants had nonoverlapping data between the baseline phase and the MEYA training phase for 6 of 8 (75%), 2 of 3 (66.7%), 9 of 10 (90%), and 5 of 7 (71.4%) of MEYA sessions, respectively. Interestingly, participants 4 and 7 had fewer than half of their MEYA sessions with nonoverlapping *MEYA-FS Change in Competence* scores (compared to baseline), despite the notable effect of MEYA training on their adherence as discussed above. Comparatively, Participant 3 exhibited consistently lower *MEYA-FS Change in Competence* scores during the MEYA phase than during baseline, mirroring his pattern of adherence scores.

The LMM for *MEYA-FS Change in Competence* scores, presented in Table 2, largely mirrors the LMM results for adherence. On average, switching from baseline to MEYA training was associated with a statistically significant increase in competence in MEYA practices. As with the adherence model, the final Hessian matrix for the initial competence model was not positive definite, and the random effect of treatment was removed from the final model, which then converged without error. As with the adherence model, exploratory LMMs were used to test for intervention effects on individual *MEYA-FS Change in Competence* items. Participants’ scores on 5 of 9 *MEYA-FS Change in Competence* items exhibited statistically significant improvement (*p*s < 0.05) from the baseline phase to the MEYA training phase (these items include Rehearsal, Cognitive, Peer Skills, Home Based Rewards, and Homework). Conversely, practitioners scores did not significantly improve on the Modeling, In-Session Reinforcement, Perspective Taking, and Goal Chart items once MEYA training began. The Exposure and Self-Management items had insufficient variance for an LMM to converge.

### Implementation Outcomes

Six of seven clinicians completed the URP-I survey, and *M*s (*SD*s) were 4.71 (0.62), 4.94 (0.31), and 4.52 (0.35) for Acceptability, Understanding, and Feasibility, respectively (one clinician was lost to follow-up and did not complete this measure). A score of 4 corresponds with “slightly agree”, and 5 corresponds with “agree”, with almost all item ratings given greater or equal to 4 (93%). Further detail on the ratings is provided in Online Resource 1 (Results).

In addition to practitioners’ self-ratings of a strong understanding of MEYA on the URP-I survey, MEYA Content Quiz (n = 6) results illustrated that practitioners improved their knowledge of MEYA implementation during their training. A repeated measures ANOVA was used to test for changes in practitioner’s mean quiz scores over this timeframe. A statistically significant increase in scores was found (*F* = 9.26, *p* = 0.03), with *M*s (*SD*s) of 10.67 (1.21), 15.67 (1.86), and 15.17 (1.17) at baseline, session 3, and post-training, respectively. On average, practitioners’ knowledge of MEYA procedures increased after training began.

#### Youth Outcomes

YTP ratings were obtained from 6 of 7 parents at baseline, and all 6 families reported at least one problem in each of the six clinical target areas addressed in MEYA (anxiety and depression, disruptive and dysregulated behavior, restricted and repetitive behavior, peer engagement, conversation and friendship, and self-care skills) with one exception: 5 of 6 families reported at least one self-care concern. Practitioners obtained parent ratings for each child’s set of YTPs at the end of the baseline phase and again at the end of the MEYA phase. Although limited in interpretability due to the variability in MEYA sessions when final ratings were taken, end-of-treatment YTP total scores were compared to last baseline session YTP total scores made by the parents of children working with Participants 1, 2, 3, 4, 5, and 7, with the following percent reductions of YTP total scores (in *n* MEYA sessions): 58.3% (in 9 sessions), -4.5% (in 11 sessions), 38.4% (in 8 sessions), 7.4% (in 10 sessions), 13.1% (in 5 sessions), and 48.9% (in 9 sessions), respectively. This same pattern was seen within the individual clinical areas, with the most consistent final-baseline to end-of-MEYA improvements seen in anxiety and depression (5 of 6 children), restricted and repetitive behaviors (5 of 6 children), peer engagement (5 of 6 children), and self-care skills (4 of 5 children), conversation and friendship (4 of 6 children) and, lastly, disruptive and dysregulated behavior (3 of 6 children). Hence, there was evidence of gains in personalized goals for most child participants.

## Discussion

This study was conducted as an initial evaluation of a free training program for practitioners working with children and youth on the autism spectrum, with a long-term goal of making high-quality psychotherapy and counseling practices accessible to any interested practitioner. In this volunteer sample of seven practitioners, results suggested that five participants measurably improved their adherence to EBPs for autistic children. Four of the seven practitioners also improved their competence in these EBPs. Preliminary youth outcomes were positive. The MEYA website may merit further research and development as a support for the implementation and dissemination of EBPs for autistic children and youth.

The adherence data in this study suggest that internet-based training in EBPs for practitioners working with autistic children has the potential to support learning even with little to no live consultation to supplement self-instruction. A substantial research literature on new medical technology illustrates that learning curves are evident in practitioner uptake of computer-mediated technology used in health interventions, with fidelity of usage improving slowly over time, even after initial training (e.g., Soomro et al., 2020). Presumably participating practitioners experienced a learning curve with MEYA. Perhaps the self-instructional resources in MEYA are nonetheless sufficient for some practitioners to learn how to implement EBPs to a certain extent during their initial utilization of this training platform.

Considering the relative expertise in behavioral and cognitive methods noted by participating practitioners, several of whom reported extensive experience in working with autistic children as well, it was notable that exposure to MEYA training still facilitated increases in adherence to practices emphasized in the MEYA session preparation materials for about 70% of the practitioners. The preliminary data on implementation outcomes suggested that practitioners were willing to try the program materials over numerous sessions, increased their understanding of the techniques, and found the program to be acceptable.

Comparatively, research has found that parent fidelity is significantly higher for parents offered internet-based training (including self-instruction) in early intervention for their children on the autism spectrum than for those who do not get internet-based training (Glenn et al., 2022). Collectively, these studies suggest that internet-based training has the potential to support adherence for some caregivers and professionals working with autistic youth. However, internet-based self-instruction did not produce universally better fidelity outcomes, likely due to several factors including variable practitioner engagement, applicability of content, and personal factors such as learning style and theoretical orientation.

Regarding the findings related to practitioner competence, fewer practitioners evidenced an increase in competence than adherence, and some observed increases were relatively attenuated (e.g., Practitioner 6). This may be because competence in delivery is slower to change than adherence (e.g., McLeod et al., 2018, 2019). Being able to repeat the steps associated with a therapeutic technique (adherence) may come faster than being able to deliver all steps with skill while attending to the competing demands for a practitioner’s attention during a session (competence). Relatively few studies have investigated how adherence and competence scores change as a result of training, suggesting essential directions for future research. In addition, research findings have not definitively established whether adherence or competence significantly impact clinical outcomes (see Collyer et al., 2020, for a review). Adherence and competence nonetheless represent established and separable metrics for assessing quality improvement (i.e., to gauge the quality of EBP implementation). When initially learning to deliver an EBP, adherence scores may provide the best indicator of uptake. In contrast, competence may be a better indicator of continued improvement. In future implementation trials of MEYA and similar EBP training supports, it will be worthwhile to monitor both adherence and competence.

Three of the practitioners in the current sample indicated that the MEYA program may be difficult to implement exactly as described in the online training. This may reflect a failure to understand the EBP principle of flexibility-within-fidelity (e.g., Kendall et al., 2008). Addressing such concerns about the usability of the MEYA training program will be important to optimize the effectiveness of MEYA in community settings. In a future paper, we plan to use mixed methods that include utilization and interview data to understand how MEYA may be optimized for use in community settings.

### Strengths, Limitations, and Alternative Designs Considered

Strengths of this study included the use of objective ratings of practitioner adherence and competence made by trained raters masked to phase (baseline vs. MEYA) as well as collection of preliminary youth and implementation outcomes. There were also several limitations of the study. We did not record the number of recruitment attempts or the number of agencies contacted in total, and a convenience sample was used, limiting the generalizability of the findings. Anecdotally, practitioners found two elements of the study to be difficult, namely, recruiting families to participate in the study alongside them (e.g., a request that could, for example, be experienced by both parties as undermining the expertise of the practitioner—a point that will be explored in our mixed methods study with this sample), and videotaping sessions (a requirement of the study that may have been uncomfortable for practitioner and family members alike in community practice settings). Many potential participants were excited about MEYA training, but in some cases were deterred by these research requirements.

Incomplete information on practitioners’ background and training prevented a more in-depth exploration of factors such as the impact of experience on outcomes; this is a limitation since years of experience with autism can facilitate greater adherence and competence. Furthermore, a multiple baseline study of this sort should be viewed as an initial exploration of practitioner fidelity, pending further research with larger samples in randomized, controlled trials. However, the study had an adequate sample size given its single case experimental design and thus helped ascertain practitioners’ response to the first version of MEYA. A more diverse and representative group of participants in the next phase of research on MEYA will also be essential. It should be noted that MEYA was not developed with the present sample of practitioners, so the lack of diversity in this study did not feed back into the intervention protocol itself. Ongoing input from a diverse group of practitioners will be critical to refining and improving MEYA. Information on the dose–response relationship between MEYA website utilization and fidelity would also have been informative; future research should address this. Lastly, there are limitations to an intervention primarily driven by caregivers’ ratings of problems and goals; children’s and teachers’ input on goals should be considered in future iterations of MEYA. The outcome assessments based on caregiver ratings in this study were promising but additional assessments of children’s outcomes will be critical in future research.

An alternative design for multiple baseline trials is to continue the baseline period until a stable baseline trend is achieved, as opposed to randomizing to varying lengths of baseline. A stable baseline can lead to less ambiguous conclusions about the treatment effect when conducting visual analysis of the data. We decided to use random assignment for two reasons. First, research has demonstrated that there may be statistical advantages to using randomization in multiple baseline designs (see Koehler & Levin, 1998) and recent guidelines for single case design studies suggest that randomization to a pre-specified baseline periods is an acceptable design approach (e.g., Ledford et al., 2023). Second, given the likelihood that some therapy episodes would end in under 10 sessions based on typical patterns in community care, it was more pragmatic to use random assignment. Nonetheless, due to the use of randomization in this study, several participants did not have an entirely stable baseline trend (and one participant only had one recorded baseline datapoint), adding some ambiguity to the interpretation of the results, particularly from the perspective of the visual analysis of the data. Similarly, many multiple baseline studies use an invariant sequence of intervention techniques to control for order effects; because a strength of MEYA is its personalized module allocation procedures, this was not possible in this study. To address this, the MEYA-FS Change scales were designed to estimate adherence and competence across the different modules so fidelity to a wide range of possible clinical content in MEYA could be compared on a relatively universal metric (McLeod et al., 2022). It is still possible that there were some order effects of different sequences of modules selected by the practitioners on the trajectory of their MEYA-FS scores. A final design element worth noting is the MEYA algorithm utilization of a cutpoint of 5 (out of 10) as a indicator of clinical need when selecting particular modules; this was a rationally selected parameter based on clinical experience and it is an empirical question whether intervention and clinical outcomes could be more effective with a different threshold score selected.

## Conclusion

The current quality of care for school-aged youth on the autism spectrum in community settings is an indicator of the science-practice gap, and this study aimed to contribute to efforts to eventually close this gap. In this study, a free internet-based training program was developed to support the implementation of efficacious EBPs for school-aged youth on the autism spectrum to overcome the twin challenges of (a) insufficient uptake and mastery of EBPs for autism among practitioners in community settings and (b) insufficient personnel and funding for traditional purveyor-based methods of dissemination of EBPs that rely on in-person expert training and supervision. Future effectiveness trials could use training platforms like MEYA to examine downstream effects on practitioners’ fidelity of implementation in their everyday psychotherapy or counseling with autistic children and on children’s outcomes. Also, an important direction for future research will be identifying factors influencing treatment fidelity. Ultimately, we hope that MEYA and similar innovations could enable widespread access to EBPs for youth on the autism spectrum in school and community settings.

## Supplementary Information

Below is the link to the electronic supplementary material.

Supplementary file1 (PDF 284 KB)
