# Supplementary material for: Practitioner Adherence and Competence in MEYA, a Free Online Self-Instruction Program in Modular Psychotherapy and Counseling for Children’s Autism-Related Clinical Needs
Source: J Autism Dev Disord. 2024 Jan 26;55(2):472–86. doi: 10.1007/s10803-023-06226-w (PMC11813990; doi:10.1007/s10803-023-06226-w)
Supplement: Supplementary file 1 — Supplementary file1 (PDF 284 KB) [file 10803_2023_6226_MOESM1_ESM.pdf]

Online Resource 1

Practitioner Adherence and Competence in MEYA, a Free Online Self-Instruction Program in Modular Psychotherapy and Counseling for Children's Autism-Related Clinical Needs

Journal of Autism and Developmental Disorders

Online Resource 1: Background

An Empirically Based Model of Practitioner Training in an Online, Self-Instructional Context

Surprisingly little research has been devoted to practitioner training and consultation in EBPs, but the adult learning literature offers relevant guidance (e.g., Soderstrom & Bjork, 2015). Drawing from this literature, we have proposed a mechanistic model of practitioner learning and mastery of EBPs (McLeod et al., 2018). Although some of the facets of this model do not apply to a self-instructional context (e.g., behavioral rehearsal with feedback), several do apply, as described below. Additionally, in a self-instructional context, design features to compensate for the lack of live interaction and feedback from an expert are critical. We adopted user-centered design (UCD; see Lyon & Koerner, 2016) as a framework for conceptualizing program elements that could enhance the effectiveness of a self-instructional format. Several key principles of adult learning and UCD served as the basis of our model of online, self-guided practitioner training; these are described below.

In the adult learning literature, observational learning (e.g., learning from modeling) tends to yield memories that are more easily retrieved when needed (e.g., Yoon et al., 2021), and multi-modal learning is generally superior to single modality learning (e.g., Taylor et al., 2005). Putting this research into practice, observations of high-quality demonstrations of a clinical intervention (e.g., videos of an expert practitioner working with a child) are likely to be an important component of a self-instructional program. Second, activities that encourage the formation of procedural knowledge, such as cognitive rehearsal, tend to enhance long-term learning (cf. McLeod et al. 2018); self-quiz activities using clinical vignettes, "what if" questions, and automated feedback may support this learning in a self-instructional context. Third, information learned recently is more easily recalled than information learned more distally (e.g., Morrison et al., 2014). This principle further highlights the advantages of providing relevant training *just before* (i.e., hours or days before) an actual intervention session to maximize recall of clinical intervention components. Fourth, when positive affect is associated with a stimulus, it is easier to remember than under other affective conditions (e.g., Anderson et al., 2006). Implementation research has suggested that practitioners tend to have positive attitudes and feelings toward EBPs that allow for practitioner choice and selection (e.g., which module to choose; which training video vignette to watch; which segment of the written manual to review by selecting between several possible weblinks; cf. Borntrager et al., 2009). This finding is consistent with models of human motivation (i.e., Self-Determination Theory; Deci & Ryan, 2012) and also has resonance with conceptualizations of the alliance in the consultation context (McLeod et al., 2018). Thus, an internet-based training platform that incorporates practitioner choice may engender positive affect in some practitioners and potentially enhance memory recall for the clinical intervention and its training elements. In short, numerous empirically-driven program characteristics may support learning during internet-based training for practitioners in community service settings.

Online Resource 1: Method

Since the onset of the development phase for MEYA, practitioners have been involved in

feedback and refinement of its training and clinical features. Therapists involved in multiple clinical trials of BIACA and SEBASTIEN have given iterative feedback for over a decade on refinement to technique and wording of the original treatment modules. Fourteen community practitioners who have contacted our team have tested beta versions of MEYA and offered suggestions on website features (placement of links, functionality of YTP tracker, kinds of examples of therapist dialogue to provide, etc.) as well. Refinement and improvement of the website, training components, and clinical materials will continue in an iterative manner as effectiveness and implementation trials continue.

Online Resource 1: Results (Supplemental Results)

URP-I. Three practitioners disagreed that the intervention could be implemented exactly as described in the online training. All practitioners rated 5 (agree) for statements regarding their ability to implement the therapeutic procedures. All agreed (one slightly) that the time required to use MEYA was reasonable. All agreed that MEYA benefited their child client (2 slightly agreed, 2 agreed, and 2 strongly agreed). All agreed that MEYA was an acceptable intervention strategy for the client's target behaviors and was effective for addressing various challenging behaviors. This response pattern suggests that MEYA may be acceptable to and feasible for some practitioners. Further research will be needed to clarify who can benefit from such training supports, and why some do and others do not.

Online Resource 1: Results (Supplemental Tables)

Online Resource 1, Table 1

Child/Youth Client Demographic and Clinical Characteristics

Measure	*n* (%)
Gender (female)	3/7 (43%)
Ethnicity/race	
Latino/a	1/7 (14%)
Caucasian	5/7 (71%)
Latino and East Indian	1/7 (14%)

Age	*M*=13.0, *SD*=2.56
WISC-V Vocabulary Scaled Score	*M*=8.39, *SD*=2.79, Range 3-12
SRS Total T-Score	*M*=80.71, *SD*=11.59

Online Resource 1, Table 2

Child/Youth Client Service and Medication Utilization

Measure	*Current n* (%)	*Previous n* (%)
Psychologist	5/7 (71%)	7/7 (100%)
Psychiatrist	1/7 (14%)	1/7 (14%)
Speech Therapy	3/7 (43%)	4/7 (57%)
Occupational Therapy	1/7 (14%)	1/7 (14%)
Social Skills	1/7 (14%)	2/7 (29%)
Applied Behavioral Analysis	0/7 (0%)	1/7 (14%)
Psychiatric Medication	3/7 (43%)	3/7 (43%)
Stimulant	1/7 (14%)	1/7 (14%)
Anti-depressants	1/7 (14%)	1/7 (14%)
Antipsychotic	1/7 (14%)	1/7 (14%)

Online Resource 1, Table 3

Clinical Modules and Corresponding Evidence-Based Practices Utilized in MEYA

**MEYA Module**	**Primary Evidence-Based Practices Utilized**
Core Skills	Self-management, perspective taking and reframing, and positive reinforcement (direct intervention and parent training, including weekly homework) presented with pivotal response features
Dysregulated and Disruptive Behavior	Core Skills (above) *plus* modeling, antecedent management, and extinction
Anxiety and Depression	Core Skills (above) *plus* systematic desensitization and pleasant activity scheduling (including exercise)
Rigid and Repetitive Behavior	Core Skills (above) *plus* exposure and response-prevention
Peer Engagement in School and the Community	Core Skills (above) *plus* modeling and systematic desensitization (focusing on school, playground, and community social contexts)
Conversation and Friendship	Core Skills (above) *plus* modeling and friendship skills training
Self-Care Skills	Core Skills (above) *plus* task analysis

Online Resource 1, Table 4

Participant Adherence Scores

Participant 1		
BL or MEYA	*Session*	*Score*
BL	1	0.31
BL	2	0.19
MEYA	3	1.19
MEYA	4	1.38
MEYA	5	0.56
MEYA	6	0.50
MEYA	7	1.19
MEYA	8	1.06
MEYA	9	0.63
MEYA	10	0.88

Participant 2		
BL or MEYA	*Session*	*Score*
BL	1	0.40
BL	2	0.63
BL	3	0.15
BL	4	0.30
BL	5	0.15
MEYA	6	0.28
MEYA	7	0.53
MEYA	8	1.08
MEYA	9	--

Participant 3		
BL or MEYA	*Session*	*Score*
BL	1	0.52
BL	2	0.92
BL	3	0.04
BL	4	0.81
BL	5	0.19
BL	6	0.04

MEYA	7	0.17
MEYA	8	0.23
MEYA	9	0.29
MEYA	10	-0.46
MEYA	11	-0.08

Participant 4		
BL or MEYA	*Session*	*Score*
BL	1	0.39
BL	2	-0.04
BL	3	0.27
BL	4	0.29
BL	5	0.50
BL	6	0.80
BL	7	0.72
BL	8	0.63
MEYA	9	2.61
MEYA	10	1.63
MEYA	11	1.45
MEYA	12	0.70
MEYA	13	0.82
MEYA	14	1.96
MEYA	15	2.04
MEYA	16	--

Participant 5		
BL or MEYA	*Session*	*Score*
BL	1	-0.08
BL	2	0.48
BL	3	-0.08
BL	4	0.33
BL	5	0.41
BL	6	0.28
BL	7	0.27
BL	8	0.47
MEYA	9	1.05
MEYA	10	--

MEYA	11	1.64
MEYA	12	1.63
MEYA	13	1.47
MEYA	14	1.08
MEYA	15	1.33
MEYA	16	1.39
MEYA	17	2.00
MEYA	18	0.59
MEYA	19	1.41
MEYA	20	--
MEYA	21	--

Participant 6		
BL or MEYA	*Session*	*Score*
BL	1	0.09
BL	2	0.31
BL	3	0
BL	4	0.22
BL	5	--
MEYA	6	1.00
MEYA	7	1.03
MEYA	8	1.56
MEYA	9	2.44
MEYA	10	2.03
MEYA	11	0.50
MEYA	12	1.91
MEYA	13	0.28
MEYA	14	--
MEYA	15	--
MEYA	16	--
MEYA	17	--

Participant 7		
BL or MEYA	*Session*	*Score*
BL	1	0
BL	2	--
MEYA	3	1.50
MEYA	4	2.13
MEYA	5	2.38
MEYA	6	--
MEYA	7	--
MEYA	8	2.25
MEYA	9	--
MEYA	10	3.00
MEYA	11	2.00
MEYA	12	--
MEYA	13	--
MEYA	14	--

Online Resource 1, Table 5

Clinician Competence Scores Per Session

Participant 1		
BL or MEYA	*Session*	*Score*
BL	1	0
BL	2	0
MEYA	3	0
MEYA	4	0.50
MEYA	5	0.50
MEYA	6	1.50
MEYA	7	0.50
MEYA	8	0.33
MEYA	9	0
MEYA	10	0.50

Participant 2		
BL or MEYA	*Session*	*Score*
BL	1	0.50
BL	2	0.20
BL	3	-0.43
BL	4	0.10
BL	5	0.10
MEYA	6	1.20
MEYA	7	0.25
MEYA	8	0.60
MEYA	9	--

Participant 3		
BL or MEYA	*Session*	*Score*
BL	1	0.25
BL	2	1.25
BL	3	0.08
BL	4	-0.33
BL	5	0.08
BL	6	-0.46

MEYA	7	0.08
MEYA	8	-1.00
MEYA	9	0
MEYA	10	0.08
MEYA	11	-1.46

Participant 4		
BL or MEYA	*Session*	*Score*
BL	1	-0.33
BL	2	--
BL	3	-0.17
BL	4	--
BL	5	-0.67
BL	6	0.50
BL	7	--
BL	8	0.17
MEYA	9	--
MEYA	10	1.17
MEYA	11	-0.17
MEYA	12	--
MEYA	13	-0.83
MEYA	14	-0.25
MEYA	15	0.42
MEYA	16	--

Participant 5		
BL or MEYA	*Session*	*Score*
BL	1	0
BL	2	0.05
BL	3	0
BL	4	-0.20
BL	5	-0.20
BL	6	0.20
BL	7	0.30
BL	8	0
MEYA	9	1.55
MEYA	10	--

MEYA	11	1.55
MEYA	12	0.80
MEYA	13	1.55
MEYA	14	1.20
MEYA	15	0.55
MEYA	16	1.80
MEYA	17	1.30
MEYA	18	-0.15
MEYA	19	1.30
MEYA	20	--
MEYA	21	--

Participant 6		
BL or MEYA	*Session*	*Score*
BL	1	--
BL	2	0.25
BL	3	--
BL	4	0
BL	5	--
MEYA	6	1.75
MEYA	7	--
MEYA	8	1.50
MEYA	9	1.50
MEYA	10	0.25
MEYA	11	0.50
MEYA	12	0.50
MEYA	13	-0.50
MEYA	14	--
MEYA	15	--
MEYA	16	--
MEYA	17	--

Participant 7		
BL or MEYA	*Session*	*Score*
BL	1	0
BL	2	--
MEYA	3	--

MEYA	4	-1.00
MEYA	5	-0.25
MEYA	6	--
MEYA	7	--
MEYA	8	0
MEYA	9	--
MEYA	10	0.25
MEYA	11	0.25
MEYA	12	--
MEYA	13	--
MEYA	14	--

Online Resource 1 References

Anderson, A. K., Yamaguchi, Y., Grabski, W., & Lacka, D. (2006). Emotional memories are not all created equal: Evidence for selective memory enhancement. *Learning & Memory*, *13*(6), 711–718. https://doi.org/10.1101/lm.388906

Borntrager, C. F., Chorpita, B. F., Higa-McMillan, C., & Weisz, J. R. (2009). Provider attitudes toward evidence-based practices: Are the concerns with the evidence or with the manuals? *Psychiatric Services*, *60*(5), 677–681. https://doi.org/10.1176/ps.2009.60.5.677

Deci, E. L., & Ryan, R. M. (2012). Self-determination theory. In P. A. M. Van Lange, A. W. Kruglanski, & E. T. Higgins (Eds.), *Handbook of Theories of Social Psychology* (pp. 416–436). Sage Publications Ltd. https://doi.org/10.4135/9781446249215.n21

Lyon, A. R., & Koerner, K. (2016). User-centered design for psychosocial intervention development and implementation. *Clinical Psychology: Science and Practice*, *23*(2), 180–200. https://doi.org/10.1111/cpsp.12154

McLeod, B. D., Cox, J. R., Jensen-Doss, A., Herschell, A., Ehrenreich-May, J., & Wood, J. J. (2018). Proposing a mechanistic model of clinician training and consultation. *Clinical Psychology: Science and Practice*, *25*(3), e12260. https://doi.org/10.1111/cpsp.12260

Morrison, A. B., Conway, A. R., & Chein, J. M. (2014). Primacy and recency effects as indices of the focus of attention. *Frontiers in Human Neuroscience*, *8*, 6. https://doi.org/10.3389/fnhum.2014.00006

Soderstrom, N. C., & Bjork, R. A. (2015). Learning versus performance: An integrative review. *Perspectives on Psychological Science*, *10*(2), 176–199. https://doi.org/10.1177/1745691615569000

Taylor, P. J., Russ-Eft, D. F., & Chan, D. W. (2005). A meta-analytic review of behavior modeling training. *Journal of Applied Psychology*, *90*(4), 692–709. https://doi.org/10.1037/0021-9010.90.4.692

Yoon, H., Scopelliti, I., & Morewedge, C. K. (2021). Decision making can be improved through observational learning. *Organizational Behavior and Human Decision Processes*, *162*(1), 155–188. https://doi.org/10.1016/j.obhdp.2020.10.011
